# Supplementary figures and images for: Burrowing crabs and physical factors hasten marsh recovery at panne edges
Source: PLoS One. 2022 Jan 5;17(1):e0249330. doi: 10.1371/journal.pone.0249330 (PMC8730443; doi:10.1371/journal.pone.0249330)

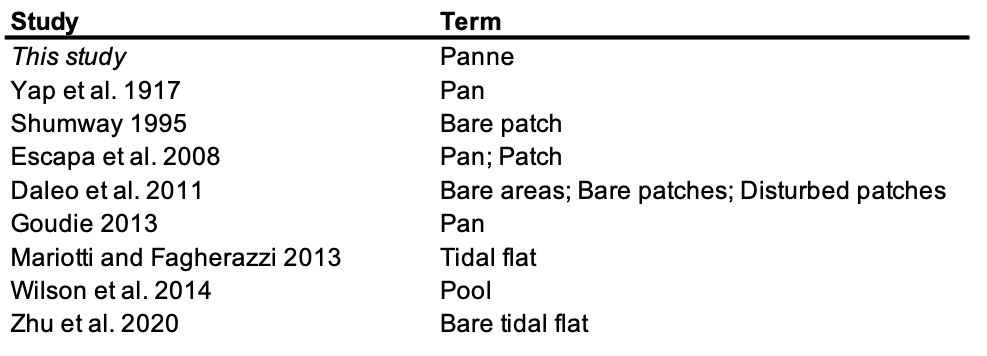

Supplement: S1 Table — All of the terms listed in the table are collectively referred to as ‘pannes’ in our study. (PNG) [file pone.0249330.s001.png]

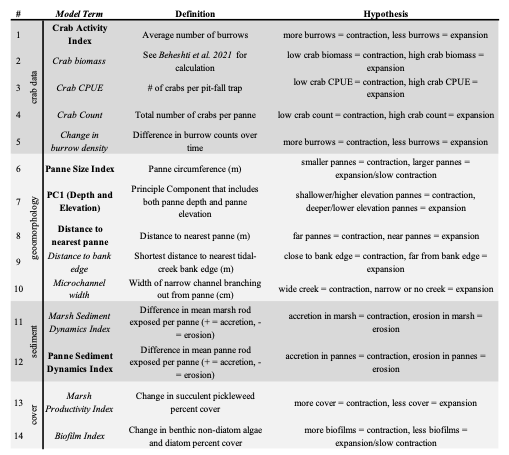

Supplement: S2 Table — Non-significant terms were excluded from the final model and are denoted in italics. Terms that were significant and included in the final model are bolded. For each term we have included the definition and hypothesis about how that factor may influence panne dynamics. Terms are organized by category (crab data, geomorphology, sediment, and cover). (PNG) [file pone.0249330.s002.png]

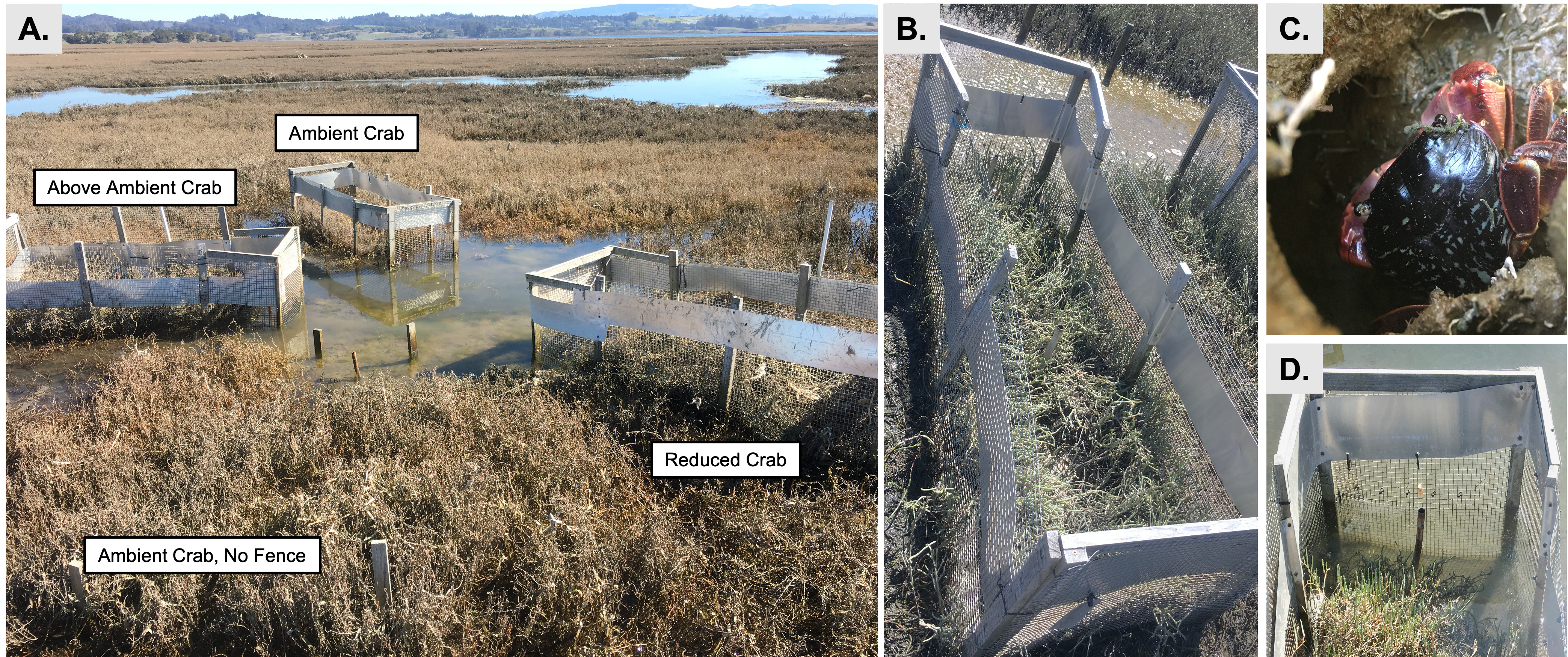

Supplement: S1 Fig — (A) Study design with treatments labeled at single block, (B) Above Ambient Crab experimental plot with the flashing installed flush to the fence wall, (C) P. crassipes crab in a burrow in one of our experimental plots and (D) Close up view of the panne rod and the zip-tie marker for the transects that run from the panne-edge to marsh-edge of the plot (See S4 Fig). (PNG) [file pone.0249330.s003.png]

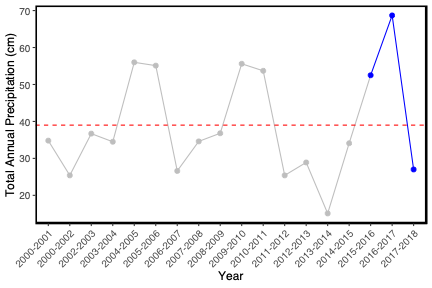

Supplement: S2 Fig — Time series of monthly precipitation (cm) from 2000–2018. The grey points represent the water years leading up to the study (2000–2015) and the blue points represent our study period (2015–2018). The dotted red line represents the long-term average annual precipitation of 39 cm. Data are from the NOAA National Estuarine Research Reserve System (NERRS). System-wide Monitoring Program. Data accessed from the NOAA NERRS Centralized Data Management Office website: http://www.nerrsdata.org/; accessed 21 September 2021. (PNG) [file pone.0249330.s004.png]

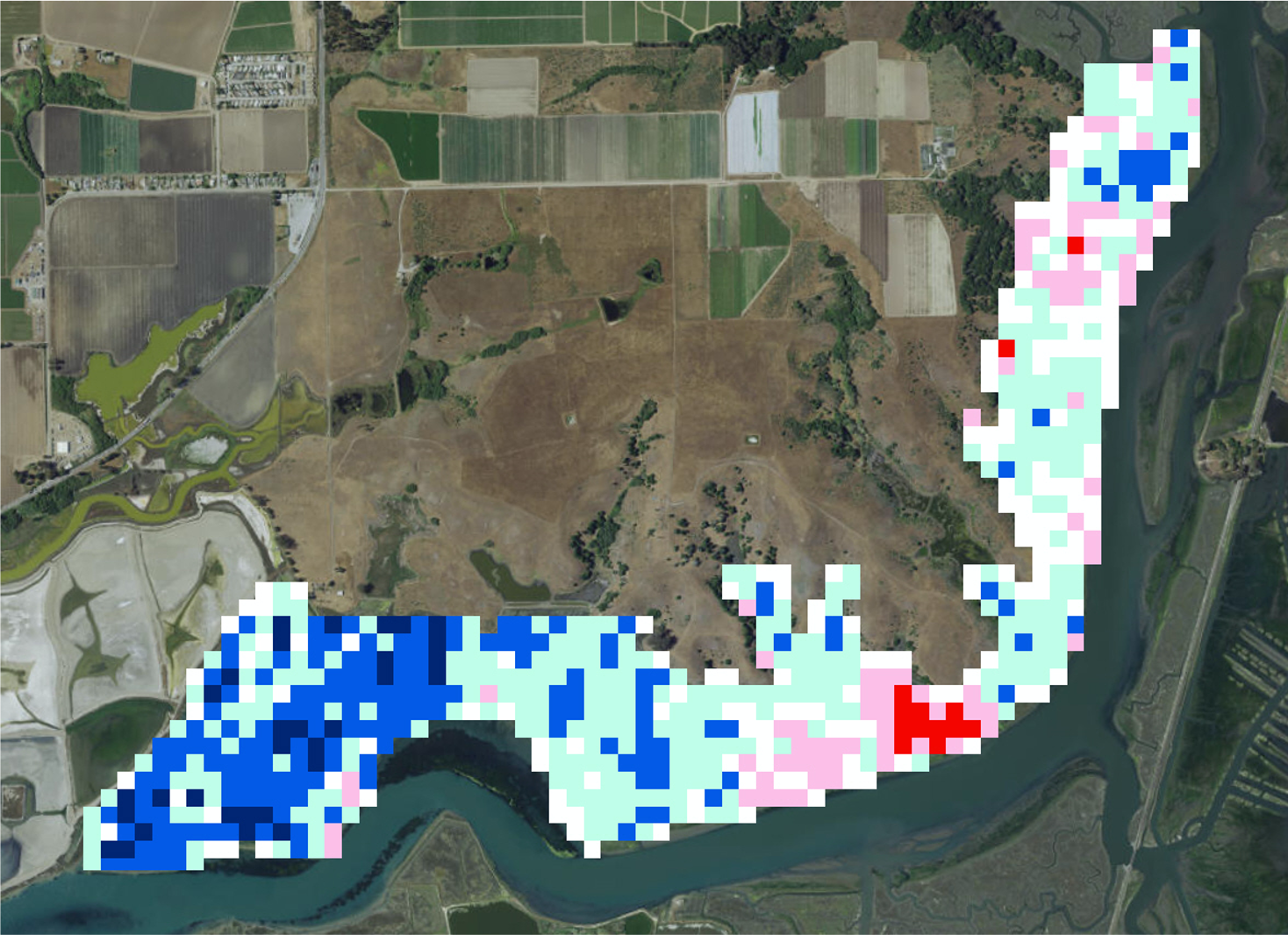

Supplement: S3 Fig — Areas where there has been net gain (blue) or loss (red) of habitat from 2004–2012. Areas of high gain or loss (dark colors) were not included in this study. NAIP imagery, Courtesy of the U.S. Department of Agriculture, Farm Service Agency. (PNG) [file pone.0249330.s005.png]

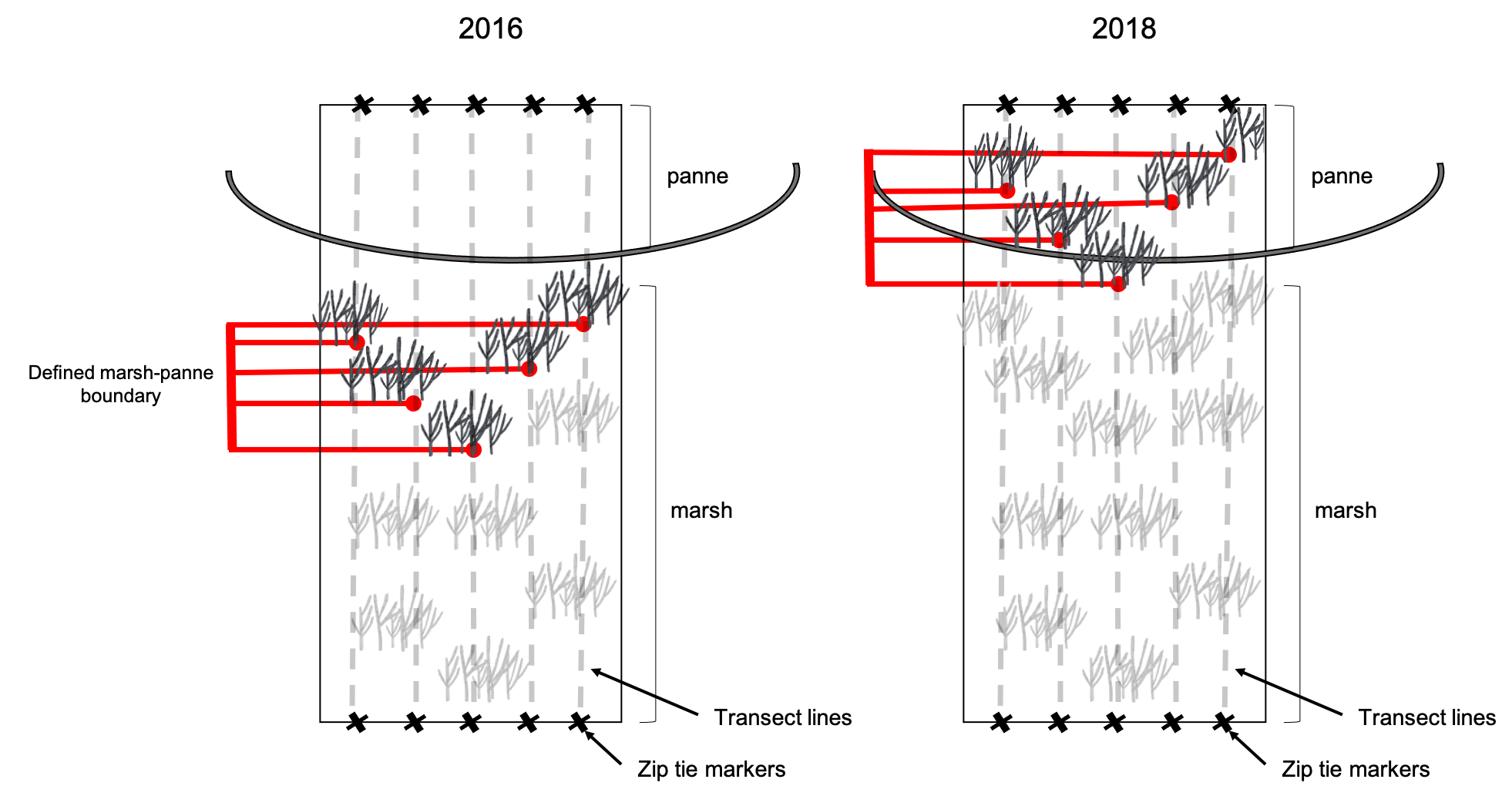

Supplement: S4 Fig — Changes to the marsh-panne boundary over time indicate either marsh colonization and panne contraction (as pictured here) or marsh dieback and panne expansion. (PNG) [file pone.0249330.s006.png]

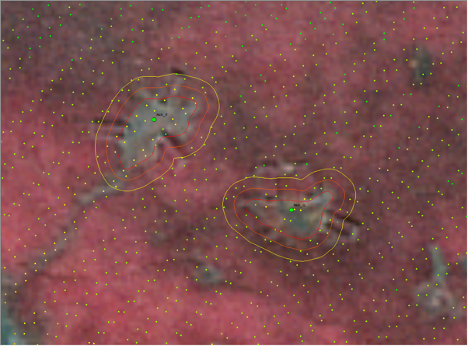

Supplement: S5 Fig — A 1 m buffer circle (shown in yellow) was used to extract the 2018 DEM cells. Raw lidar points (shown in the image as green points) were not used since they were not particularly well-spaced. The DEM uses an interpolation between the points, and thus was reliable at representing the marsh-panne boundary and the pannes themselves. Courtesy of the U.S. Geological Survey. (PNG) [file pone.0249330.s007.png]

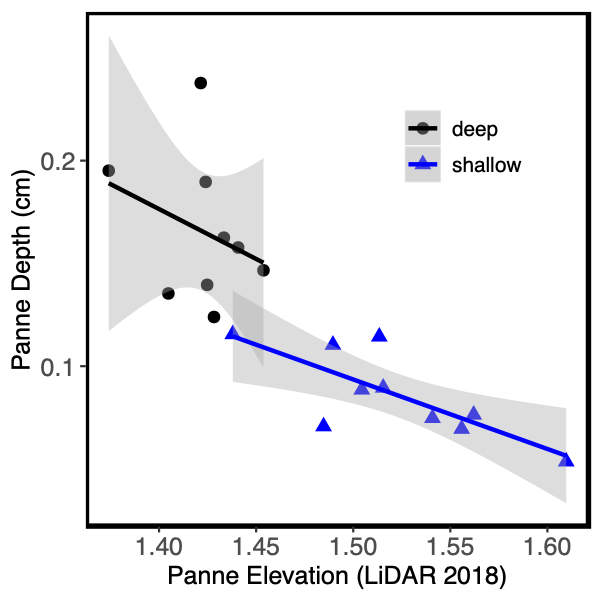

Supplement: S6 Fig — This inverse correlation led to the development of a Principle Component (PC1 (Depth and Elevation)). Deep pannes are indicated by black circles and shallow pannes by blue triangles. Reported in the top left corner of the plot is the R2 and plotted regressions include the 95% C.I. (PNG) [file pone.0249330.s008.png]

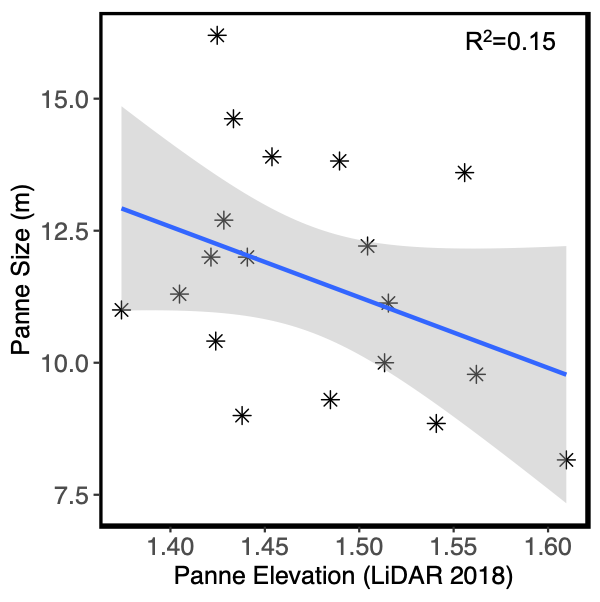

Supplement: S7 Fig — As panne elevation increases, size decreases (R2 = 0.15). Similar patterns were observed in Escapa et al. [31] (See Fig 3)—panne size (‘Patch diameter’; Escapa et al. 2015) decreased as elevation increased. The lower elevation edge for pickleweed in Elkhorn Slough is ~1.20 m NAVD 88 (C. Endris, unpublished data), our study did not extend lower than 1.37 m NAVD 88. (PNG) [file pone.0249330.s009.png]

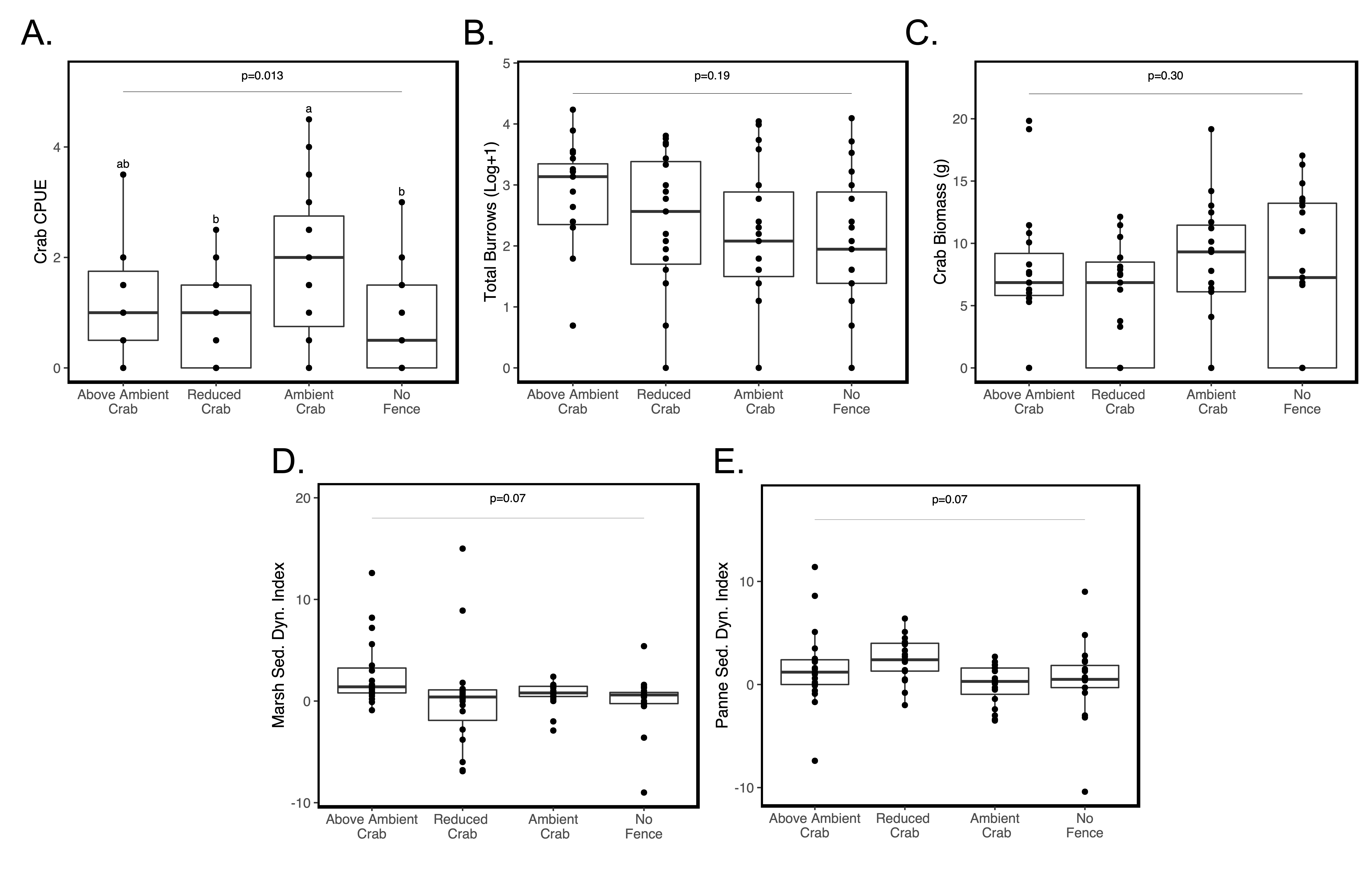

Supplement: S8 Fig — (A) crab CPUE, (B) burrow and (C) crab biomass data from 2018 plotted by treatment. To demonstrate that there was little evidence of caging effects on drivers related to sediment dynamics and/or crabs, we have shown the (D) panne (F3,72 = 2.46, p = 0.07) and (E) marsh (F3,72 = 2.45, p = 0.07) rod data. (PNG) [file pone.0249330.s010.png]

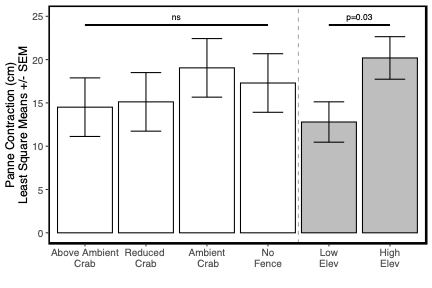

Supplement: S9 Fig — There was no significant effect of treatment on marsh-panne boundary movement, or the rate of panne contraction (F3,3 = 0.38, p = 0.77). There was an elevation effect, with significantly greater rates of panne contraction for high versus low elevation pannes (F1,1 = 4.77, p = 0.03). Elevation data is collected at the panne level, treatment data is collected at the sub-sample level and averaged across all pannes. There was no treatment*elevation effect. Plotted is the least square mean ± standard error. Treatment data (white bars) is plotted to the left of the dotted gray line and elevation data (gray bars) is plotted to the right of the dotted gray line. (PNG) [file pone.0249330.s011.png]
