# Supplementary material for: Burrowing crabs and physical factors hasten marsh recovery at panne edges
Source: PLoS One. 2022 Jan 5;17(1):e0249330. doi: 10.1371/journal.pone.0249330 (PMC8730443; doi:10.1371/journal.pone.0249330)
Supplement: S1 Text — Methods and results are described in detail below. We designed a two-year field study to determine whether crabs affected salt panne dynamics and whether such effects varied by elevation. (DOCX) [file pone.0249330.s013.docx]

**Supplemental Information**

Beheshti, K.M., C. Endris, P. Goodwin, A. Pavlak, and K. Wasson. Physical and biological factors influence panne dynamics in a California estuary.

**Crab experiment**

To test whether crabs had an effect on salt panne dynamics and whether such effects varied by elevation, we conducted the following experiment from 2016-2018 at the 20 pannes. One of the pannes was discarded from the analyses because it was adjacent to a large seagrass meadow, resulting in extensive year-round wrack accumulation (resulting in final n=19). The key response variable we focused on was movement of the panne edge (transition from vegetated to unvegetated). Within each panne we applied four experimental treatments: (i) reduced crab densities (full fences where crab density was continually reduced using pit-fall traps), hereafter referred to as ‘Reduced Crab’, (ii) above ambient crab densities (full fences where crab density was kept above ambient densities through regular addition of individuals into the experimental plot area), hereafter referred to as ‘Above Ambient Crab’, (iii) ambient crab densities with fence (lifted fences where crabs were allowed to move in and/or out of the experimental plot area), hereafter referred to as ‘Ambient Crab’, and (iv) ambient crab densities without fence (experimental plot area marked by wooden posts), hereafter referred to as ‘No Fence’ (S1 Fig). The roots were severed using a shovel (30 cm deep x 1 cm wide) in all plots in order to prevent exchange between our experimental plots and the surrounding marsh. Each of the experimental plots was 1.5 x 0.5 m with ~0.25 x 0.5 m of plot inserted into the panne itself, and the remaining 1.25 x 0.5 m in the marsh (S2A Fig). Approximately 16% of each experimental plot was within a panne. The fences were built using 19-gauge 1.3 cm x 123 cm x 30.5 cm galvanized hardware cloth attached with staples to 1 m-long wood posts. The wire-mesh fencing around the Above Ambient Crab and Reduced Crab plots extended into the substrate ~10 cm to prevent crabs from burrowing in or out of the experimental plot area. The fence wall of the Ambient Crab plots was lifted ~5 cm from the marsh substrate to enable the crabs free access to move in and out of the plots. To keep crabs in the Above Ambient Crab plots, aluminum flashing was installed flush to the interior fence wall to prevent crabs from escaping by climbing up and out of the fence (crabs were unable to grip the flashing). To account for the potential confounding effects of the flashing, it was installed on all fenced plots. To keep crabs from entering the Reduced Crab experimental plots, flashing was installed on the outside of the fence wall and flashing was installed on the inside of Ambient Crab plots to mimic the Above Ambient Crab plots. Fences were cleaned regularly (once per month from November-March, twice per month from April-October) to remove any algal wrack deposited on the fence walls or inside the plots during high tides. This was done to remove any potential confounding factors related to algal wrack that may have been disproportionately attracted to fenced plots.

To measure ambient crab densities and set Above Ambient Crab treatment levels, we conducted a 24 hr crab trapping effort at the start of the study in 2016 using the two pit-fall traps installed flush to the sediment surface in the back marsh-end of each of the eighty experimental plots in each panne. Pit-fall traps (i.e. tennis ball cans, 3.5 cm diameter and 20.5 cm depth with holes at the base for drainage) were installed in all experimental plots to account for the potential effect of installing traps in the experimental plot area and remained capped except during our 24 hr crab trapping efforts (August 2016, March 2017, August 2018). Above Ambient Crab densities was set at one standard deviation above the mean, or 8 crabs. To maintain the Reduced Crab treatment, we continually trapped crabs, removing them from the experimental plot area using permanently un-capped pit-fall traps throughout the entire duration of the study. Pit-fall traps in the Reduced Crab plots were emptied as needed, typically monthly. Burrow density (0.75 m^2^) data was collected in each plot (n=80) annually.

Elevation data was extracted after the pannes were selected. Pannes were selected on several factors including approximate relative elevation, plant community, substrate firmness, the degree of consolidation of the sediment etc. Using a LiDAR Digital Elevation Model (2018), we extracted the elevations for all nineteen pannes that were used in analyses. We set the low elevation cut-off at 1.45 m NAVD 88. This cut-off was chosen because it allowed for somewhat even replicates by elevation (low, n=10; high, n=9). We acknowledge that a more appropriate cut-off would have been ~1.53 m which is approximately the Mean High Water (MHW) elevation of Elkhorn Slough. Low elevation blocks ranged from 1.37 to 1.45 m NAVD 88 and high elevation blocks ranged from 1.48 to 1.61 m NAVD 88.

To assess whether pannes expanded or contracted over the study period and at what rate, we installed five permanent transect line markers within each of the plots, with the exception of unfenced plots which had three permanent transect line markers. Zip ties were used to mark the longitudinal start and end of each transect to ensure we were surveying the same points over time. We could not use the zip tie method to mark the transects in the unfenced plots since there was no fence wall to secure the zip ties to. Instead, we used the wood posts and the rods as our permanent transect markers. During surveys, each of the transect lines was resurveyed and the last rooted vegetation along each transect line was recorded. Surveys were conducted annually from 2016 to 2018 (S4 Fig). To quantify contraction or expansion we calculated the average “marsh-panne boundary” difference per panne between 2016 and 2018. A positive value meant marsh colonization and panne contraction and a negative value meant marsh dieback and panne expansion. Our hypotheses for each of the parameters and indices outlined below can be found in Table 1 and S2 Table.

To test for treatment effects on panne contraction or expansion we first ran a mixed model with Block(Relative Elevation Category) as a Random Effect. After detecting no effect of Block(Relative Elevation Category) we removed it from the model and ran a two-way ANOVA with four levels (Above Ambient Crab, Ambient Crab, Reduced Crab, No Fence) of factor “Treatment” and two levels (High and Low) of factor “Relative Elevation Category”. We then tested for differences in crab CPUE and burrow density by treatment and elevation using a full factorial ANOVA with four levels (Above Ambient Crab, Ambient Crab, Reduced Crab, No Fence) of factor “Treatment” and two levels (High and Low) of factor “Relative Elevation Category”. We used the same approach to test for treatment and elevation differences in crab biomass, since previous studies showed that crab biomass was a stronger predictor of crab effects than crab CPUE [1]; fewer larger crabs are likely to have a stronger engineering effect than many small crabs.

Overall, we failed to manipulate crab or burrow densities as initially designed. We found no consistent reduction in crabs (S8A Fig) or burrows (S8B Fig)in the Reduced Crab treatments, and no significant increase in the Above Ambient treatments, relative to the Ambient and No Fence treatment. At our last sampling (August 2018), differences in crab CPUE by treatment were inconsistent with our study design, verifying that we were unable to manipulate crab densities as initially designed (ANOVA; F_3,72_=3.85, p=0.013). According to Tukey’s HSD post-hoc test, crab CPUE was not significantly different when comparing Ambient and Above Ambient Crab treatments and Above Ambient Crab was not significantly different from other treatments (Reduced Crab and No Fence). Additionally, we failed to detect an effect of treatment, elevation, or treatment*elevation on panne contraction or expansion (S9 Fig). We found no effect of treatment, elevation, or treatment*elevation on crab biomass, providing additional evidence that our experimental manipulation failed and that the various designs of fenced enclosures did influence key crab parameters (S8C Fig). To further demonstrate that there was no detectable artifact of enclosures and to provide additional justification for using the four treatments as sub-samples to characterize each of the 19 pannes, we ran an ANOVA to test whether there was an effect of treatment, elevation, or treatment*elevation on panne and marsh sediment dynamics, as measured by the rods (See Main Text). We found there to be no significant effect of treatment, elevation, or treatment*elevation on patterns of erosion or accretion in the panne or marsh area of the plots (S8D-E Fig). All statistical analyses were performed in R software (R Core Team, 2016).

1. Beheshti KM, Wasson K, Angelini C, Silliman BR, Hughes BB. Long-term study reveals top-down effect of crabs on a California salt marsh. Ecosphere. 2021; 12(8): e03703.

2. R Core Team. R: A Language and Environment for Statistical Computing, Vienna, Austria. 2016. Available at: [https://www.R-project.org/](https://www.r-project.org/)
